# Intestinal Gastrin/CCKBR (Cholecystokinin B Receptor) Ameliorates Salt-Sensitive Hypertension by Inhibiting Intestinal Na^+^/H^+^ Exchanger 3 Activity Through a PKC (Protein Kinase C)-Mediated NHERF1 and NHERF2 Pathway

**DOI:** 10.1161/HYPERTENSIONAHA.121.18791

**Published:** 2022-06-08

**Authors:** Xiaoliang Jiang, Yunpeng Liu, Xin-Yang Zhang, Xue Liu, Xing Liu, Xianxian Wu, Pedro A. Jose, Shun Duan, Fu-Jian Xu, Zhiwei Yang

**Affiliations:** NHC Key Laboratory of Human Disease Comparative Medicine (The Institute of Laboratory Animal Sciences, CAMS&PUMC), National Human Diseases Animal Model Resource Center, Beijing Engineering Research Center for Experimental Animal Models of Human Critical Diseases, P.R. China (X.J., Y.L., Xue Liu, Xing Liu, X.W., Z.Y.).; Key Lab of Biomedical Materials of Natural Macromolecules (Beijing University of Chemical Technology), Ministry of Education, Beijing Laboratory of Biomedical Materials, Beijing Advanced Innovation Center for Soft Matter Science and Engineering, Beijing University of Chemical Technology, P.R. China (X.-Y.Z., S.D., F.-J.X.).; Department of Pharmacology and Physiology (P.A.J.), The George Washington University School of Medicine and Health Sciences, Washington, DC.; Division of Kidney Diseases and Hypertension, Department of Medicine (P.A.J.), The George Washington University School of Medicine and Health Sciences, Washington, DC.

**Keywords:** blood pressure, cholecystokinin, gastrins, intestines, sodium

## Abstract

**Background:**

The present study directly tested the crucial role of intestinal gastrin/CCKBR (cholecystokinin B receptor) in the treatment of salt-sensitive hypertension.

**Methods:**

Adult intestine-specific *Cckbr*-knockout mice (*Cckbr*^fl/fl^ *villin-Cre*) and Dahl salt-sensitive rats were studied on the effect of high salt intake (8% NaCl, 6–7 weeks) on intestinal Na^+/^H^+^ exchanger 3 expression, urine sodium concentration, and blood pressure. High-salt diet increased urine sodium concentration and systolic blood pressure to a greater extent in *Cckbr*^fl/fl^ *villin-Cre* mice and Dahl salt-sensitive rats than their respective controls, *Cckbr*^fl/fl^ *villin* mice and SS13^BN^ rats. We constructed gastrin-SiO_2_ microspheres to enable gastrin to stimulate specifically and selectively intestinal CCKBR without its absorption into the circulation.

**Results:**

Gastrin-SiO_2_ microspheres treatment prevented the high salt-induced hypertension and increase in urine Na concentration by inhibiting intestinal Na^+/^H^+^ exchanger 3 trafficking and activity, increasing stool sodium without inducing diarrhea. Gastrin-mediated inhibition of intestinal Na^+/^H^+^ exchanger 3 activity, related to a PKC (protein kinase C)-mediated activation of NHERF1 and NHERF2.

**Conclusions:**

These results support a crucial role of intestinal gastrin/CCKBR in decreasing intestinal sodium absorption and keeping the blood pressure in the normal range. The gastrointestinal administration of gastrin-SiO_2_ microspheres is a promising and safe strategy to treat salt-sensitive hypertension without side effects.

**Novelty and Relevance**

**What Is New?**

Our study first showed that intestinal CCKBR (cholecystokinin B receptor) is important in the regulation of sodium balance and blood pressure.
Our study shows for the first time that gastrin-SiO_2_ microspheres, via intestinal CCKBR, inhibit Na^+^/H^+^ exchanger 3-mediated intestinal sodium absorption without causing diarrhea, and is therefore a potential clinical approach in the treatment of salt-sensitive hypertension.

**What Is Relevant?**

Intestinal CCKBR deficiency contributes to the pathogenesis and maintenance of salt-sensitive hypertension, but the cellular and molecular mechanisms remain to be determined.
Gastrin-SiO_2_ microspheres maintaining intestinal sodium metabolism by inhibiting Na^+^/H^+^ exchanger 3 activity via reducing cell surface Na^+^/H^+^ exchanger 3 protein through a NHERF1-NHERF2 and phospholipase C/PKC (protein kinase C) pathway.

**Clinical/Pathophysiological Implications?**

Gastrin-SiO_2_ microspheres, given by gavage, ameliorated salt-sensitive hypertension and organ damage by partial inhibition of Na^+^/H^+^ exchanger 3 activity to prevent inappropriate intestinal sodium absorption, without causing diarrhea and is a promising clinical approach in the treatment of salt-sensitive hypertension.
Gastrin-SiO_2_ microspheres stimulate CCKBR only in the intestines and decrease not only intestinal sodium absorption but also the risk of inflammation and cancer, enhancing their biocompatibility and safety, which might be promising for future clinical application.

Hypertension is a multifunctional disorder resulting from the interaction of the environment, genetics, epigenetics, and behavior, among which high salt intake is a common risk factor.^1^. Excessive sodium consumption (defined by the World Health Organization as >2 g sodium (5 g NaCl) per day^2^ increases blood pressure (BP) and causes hypertension, cardiovascular complications,^1,3^ and chronic kidney disease.^4^. However, several cohort studies^4–6^ and meta-analyses^7,8^ of such studies have shown that the relationship between sodium intake and BP or poor prognosis is not linear, but rather a J-shaped curve. These studies undermine the traditional view that the lower the sodium intake, the better is the overall health. The challenge stands on the way of dietary sodium restriction: the worldwide sodium intake ranges from 3.5 to 5.5 g/d (corresponding to 8.8–13.8 g of salt [NaCl] per day), which is much higher than the WHO recommendation. Therefore, interventions to inhibit the intestinal absorption of sodium to mitigate the deleterious consequences of inappropriate salt intake may be a novel therapy for hypertension.

In mammals, most of the orally ingested sodium is absorbed by the gut, primarily by the small intestines.^9,10^. Na^+^/H^+^ exchanger 3 (NHE3) at the intestinal brush border accounts for the majority of sodium absorbed by the intestines both in the basal state and in the late postprandial period.^11^. Angiotensin II–induced hypertension is attenuated in mice with global deletion of *Nhe3* (*Nhe3*^−/−^)^12^ and mice with transgenic rescue of the *Nhe3* gene (tg*Nhe3*^−/−^) in the small intestines.^13–16^. Accordingly, tenapanor and SAR218034, inhibitors of NHE3 activity, were designed to reduce the high BP in rodents and humans. However, the clinical trials of tenapanor^15^ and SAR218034^17^ were discontinued, because of the heavy diarrhea. Rieg et al^18^ also reported that small intestine-specific *Nhe3* knockout mice died within a few days after birth with no adult survival. Therefore, partially inhibiting NHE3 may be a way to decrease BP without or with minimal side effects.

Gastrin is a peptide hormone secreted by G cells in the stomach and duodenum, which induces acid secretion^19–21^. Gastrin can also regulate sodium balance and BP^22,24^ via its receptor, CCKBR (cholecystokinin B receptor) by inhibiting the activities of renal Na^+^/K^+^-ATPase^24,25^ and NHE3.^25^ We have reported that global knockout of gastrin (*Gast*)^26^ or *Cckbr*^27^ in mice causes salt-sensitive hypertension. Pharmacological inhibition of intestinal NHE3 decreases the BP of spontaneously hypertensive-obese rats.^28^ Therefore, we hypothesize that intestinal CCKBR, stimulated by gastrin, may play an important role in the intestinal sodium absorption and BP regulation by inhibiting intestinal NHE3 activity. However, increased circulating gastrin levels may promote cancer.^29^ We designed and constructed gastrin-SiO_2_ microspheres with the diameter of 70 μm to prevent its absorption in gastrointestinal tract.and determined if intestinal CCKBR, independent of renal CCKBR, can regulate BP.

## Materials and Methods

The data that support the findings of this study are available from the corresponding author upon reasonable request. Details on the animal and synthesis and characterizations of SiO_2_ microspheres and molecular assays are in the Supplemental Material.

## Results

### CCKBR Protein Expression in the Intestine of Human and Mouse

**Figure 1.**
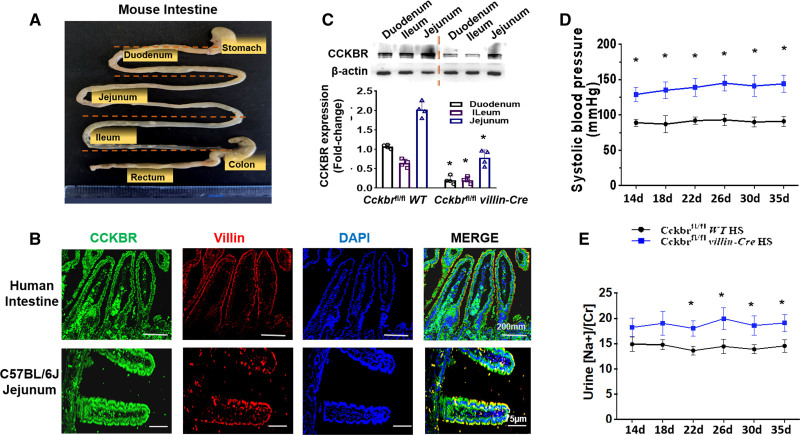
**Intestinal CCKBR (cholecystokinin B receptor) is involved in the regulation of sodium balance and blood pressure (BP). A**, Divisions of mouse gastrointestinal tract. **B**, CCKBR protein expression in 3 segments of the intestines in *Cckbr^fl/fl^* (wild type [WT]) mice and *Cckbr*^fl/fl^ *villin-Cre* (knockout [KO]) mice. **C**, Immunofluorescence of Alexa Fluor 488-labeled CCKBR (green), Alexa Fluor 568-labeled villin (red) and DAPI(blue) in human intestine and C57BL/6J mouse jejunum. **D**, Systolic BP was measured by telemetry and the data were analyzed by Acqknowledge 5.0 software. **E**, Urine sodium concentration was measured by flame photometry (*P<0.05 vs *Cckbr^fl/^f^l^* WT+high-salt (HS), 1-way ANOVA, Tukey test).

CCKBR is expressed in the renal proximal tubule and intestinal cells.^30^ Human protein atlas (https://www.proteinatlas.org) was used to retrieve the data on the expression of CCKBR in human tissues. Analyses of 3 databases, human protein atlas, genotype-tissue expression, and functional annotation of the mammalian genome 5, showed that the mRNA expression of *Cckbr* was highest in the duodenum, relative to other segments of the intestines (Figure S3). We, then, quantified the CCKBR protein expression in the different segments of mouse small intestine, divided into duodenum, jejunum, and ileum (Figure 1A). We found that CCKBR expression was highest in the jejunum, followed by the duodenum, and least in the ileum in wild-type mice (Figure 1B). Immunofluorescence study showed that CCKBR colocalized with villin and is mainly expressed at the microvillar brush border membrane of human intestine and mouse jejunum (Figure 1C).

### Intestine-Specific Knockout of *Cckbr* Increases BP and Urine Na Concentration

Western blot showed that intestinal CCKBR expression was markedly lower in *Cckbr*^fl/fl^ *villin-Cre* mice than *Cckbr*^fl/fl^ WT mice (Figure 1B). However, there was no difference in renal CCKBR expression (*P*>0.05) in these 2 groups of mice (Figure S4), indicating intestinal-specific deletion of *Cckbr*. The BP from the carotid artery was measured continuously by radiotelemetry in conscious mice. *Cckbr*^fl/fl^ *villin-Cre* mice had increased BP (*P*>0.05) but normal urine Na/Cr on normal-salt (0.49% NaCl) diet (Figure S5). Systolic BP in *Cckbr*^fl/fl^ *villin-Cre* mice increased by the second week (129±10 mm Hg) and remained elevated until the fifth week (142±12 mm Hg) of high-salt (4% NaCl) diet. These BPs were significantly higher than those in WT mice (89±3–91±6 mm Hg; Figure 1D). As shown in Figure 1E, *Cckbr*^fl/fl^ *villin-Cre* mice had higher urine Na concentration (18.22±1.85–19.10±1.67 Na+/Cr) than WT mice (14.90±1.41–14.56±1.26 Na+/Cr) from the second to the fifth week of high-salt diet.

Chronic high BP causes organ damage in the cardiovascular system.^31^ On normal-salt diet the expressions of the organ injury markers, MMP (matrix metalloproteinase)-9 and MMP-2, were higher in the kidneys of *Cckbr*^fl/fl^ *villin-Cre* mice than WT mice (*P*<0.05; Figure S6A). The high-salt diet did not increase further the elevated renal MMP-9 and MMP-2 in *Cckbr*^fl/fl^ *villin-Cre* mice but increased MMP-9 expression in WT mice to the same level as that observed in *Cckbr*^fl/fl^ *villin-Cre* mice. No obvious pathological differences were observed in the sections of the kidney and heart between these 2 mice groups (Figure S6B). Serum biochemical parameters, including liver function (alanine aminotransferase [ALT] and AST [aspartate transaminase]) and renal function (urea nitrogen and uric acid) and cardiovascular-related tests (LDL-C [low-density lipoprotein cholesterol] and CK [creatine kinase]) were markedly increased in high salt-fed *Cckbr*^fl/fl^ *villin-Cre* mice. These were minimally increased in WT mice (Figure S6C) except for serum ALT which was markedly increased by high-salt diet in WT mice.

### Gastrin-SiO_2_ Microspheres Specifically Stimulate Small Intestinal CCKBR

**Figure 2.**
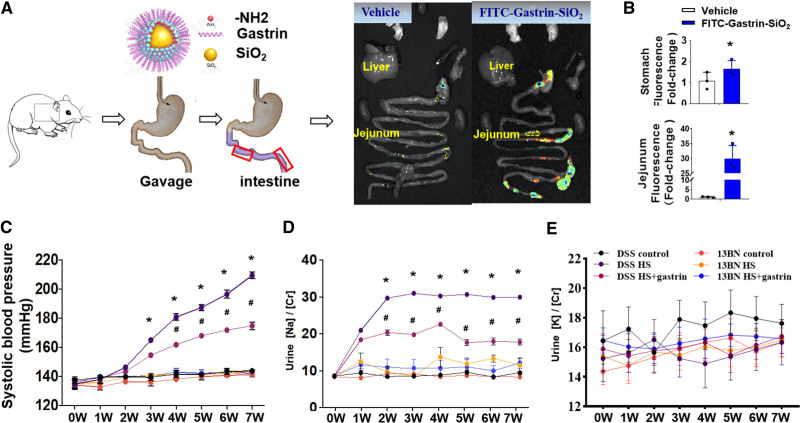
**Gastrin-SiO_2_ microspheres attenuates the sodium-induced increase in blood pressure (BP). A**, Protocol for gastrin-SiO_2_ microspheres generation and intestinal location in vivo; gastrin-SiO_2_ microspheres location determined by fluorescence emission tomography. **B**, Immunofluorescence of stomach and jejunum in the 2 groups of mice (n=3/group, *P<0.05 vs control). **C** and **D**, Gastrin-SiO_2_ microspheres ameliorated the high salt (HS)-elevated systolic BP and urine Na concentration in Dahl salt-sensitive rats fed normal (N, 0.49% NaCl) or HS (8% NaCl) diets (*P<0.05 vs Dahl salt-sensitive control [normal-salt diet], # vs Dahl salt-sensitive + HS diet, 1-way ANOVA, Tukey test). **E**, Urine potassium excretion was not different among the groups. FITC indicates fluorescein isothiocyanate.

Gastrin could regulate sodium balance by inhibiting sodium transport in the gut and the kidney.^22–25^ Gastrin-SiO_2_ microspheres were designed to work in the intestine but not in the stomach (Figure 2A). The intestinal fluorescence was visually stronger in fluorescein isothiocyanate (FITC)-gastrin-SiO_2_-gavaged mice than control mice, especially in the jejunum and ileum, with less fluorescence in the latter that in the former (Figure 2A). The signals in the stomach which may be due to spontaneous luminescence of chyme. The quantified luminescence in the jejunum was much greater in gastrin-SiO_2_-FITC than gastrin-SiO_2_-vehicle treated mice (Figure 2B). We also constructed fluorescent dye-conjugated gastrin (not conjugated to SiO_2_). Figure S7A and S7B showed strong signals in the stomach in the FITC-gastrin group, with no signal in the intestines. By contrast, the signals in the FITC-gastrin-SiO_2_ microspheres group were concentrated in the jejunum (Figure S7C), which demonstrated that without microspheres, gastrin would bind to CCKBR in the stomach, such that there may not be enough gastrin to activate intestinal CCKBR. Time-dependent fluorescence of FITC-labeled gastrin-SiO_2_ microspheres was measured (Figure S8). There was strong signal in jejunum and other intestinal segments at 8-hour postgastrin-SiO_2_ microspheres administration, which was not significantly decreased until 24-hour postadministration. Gastrin was not detected in the serum in either the vehicle- or gastrin-SiO_2_ microspheres-treated group, indicating that gastrin in gastrin-SiO_2_ microspheres was not absorbed from the intestines and released into the circulation (Figure S1). Therefore, gastrin-SiO_2_ microspheres were used to stimulate specifically and selectively intestinal CCKBR in Dahl salt-sensitive rats and the daily gavage was sufficient for intestine CCKBR activation.

### Gastrin-SiO_2_ Microspheres Treatment Mitigates Salt-Sensitive Hypertension

Conscious Dahl salt-sensitive rats had a progressive increase in systolic BP, measured by telemetry, after the second week of high-salt (8% NaCl) diet; the systolic BP of the control rats fed normal-salt (0.49% NaCl) diet was not altered during the 7-week period of observation (Figure 2C). Gastrin-SiO_2_ microspheres treatment attenuated the high salt-induced increase in systolic BP (164.8±4.2 versus 154.5±4.6 mm Hg) from the fourth week to the seventh week (209.3±5.8 versus 174.7±6.3 mm Hg; *P*<0.05). By contrast, there was no effect of the high-salt diet on systolic BP in SS13^BN^ rats (Figure 2C). Urinary sodium (Figure 2D; *P*≤0.05) excretion, which was elevated by the high-salt diet in Dahl salt-sensitive rats, was decreased by gastrin-SiO_2_ microspheres treatment. Urinary potassium excretion was not different among the groups (Figure 2E).

Western blotting showed that the elevated MMP-9 and MMP-2 expressions in the kidney of Dahl salt-sensitive rats (high salt group) were downregulated by gastrin-SiO_2_ microsphere treatment (Figure S9A and S9B). The food intake (g/wk) and body weight (g) were similar among the 3 groups (Dahl salt-sensitive rats fed normal-salt (control) diet, high-salt, or high-salt and gastrin-SiO_2_ microspheres (Tables S1 and S2). Renal histopathologic staining and plasma biochemical parameters, except for serum LDL-C, indicated that gastrin-SiO_2_ microsphere treatment protected the organs (kidney, heart, and liver) from damage (Figures S9C and S10).

It is also critical to determine if there are unfavorable effects of gastrin-SiO_2_ microspheres treatment because gastrin stimulation may take part in colon or stomach carcinogenesis by increasing proliferation and information in tumor cells.^30–33^ As shown in Figure S11, the colon cancer promoting markers (cancerantigen 199, prostate-specific antigen, and carcino-embryonic antigen) were not affected by high-salt diet or gastrin-SiO_2_ microsphere treatment. The mRNA expressions of inflammatory factors (TNF [tumor necrosis factor]-α, MCP [monocytechemoattractantprotein]-1, MCP-2, IL [interleukin]-1β, IL-6, and NF-κB [nuclear factor-κB]) were increased in the high-salt group (*P*<0.05) but not aggravated by gastrin-SiO_2_ microspheres treatment. These results indicated that gastrin-SiO_2_ microspheres are biocompatible without obvious adverse side effects and could be used to treat salt-sensitive hypertension and its complications.

### Gastrin/CCKBR Decreases NHE3 Expression and Activity in Intestinal Brush Border Membrane

**Figure 3.**
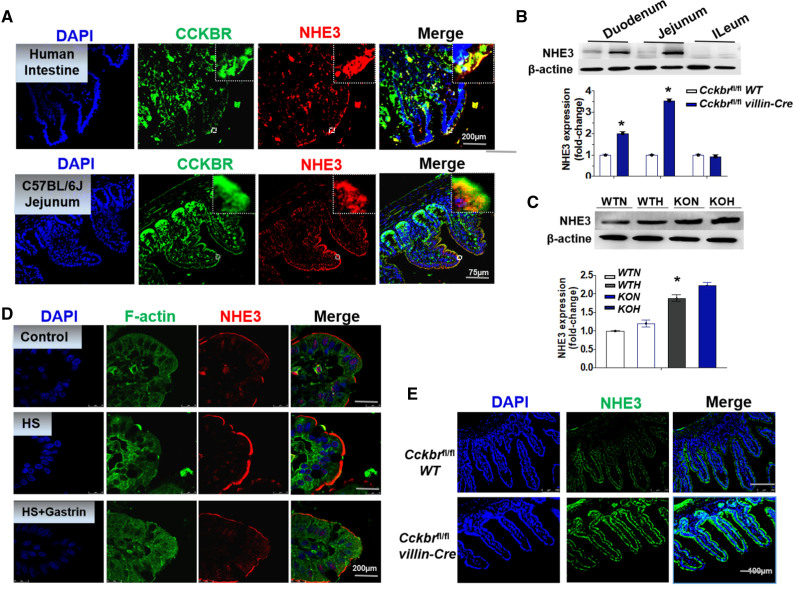
**Gastrin/CCKBR (cholecystokinin B receptor) decreases Na+/H+ exchanger 3 (NHE3) expression and activity in intestinal brush border membrane. A**, Immunofluorescence of Alexa Fluor 488-labeled CCKBR (green), and Alexa Fluor 568-labeled NHE3 (red) in human intestine (scan bar is 200 μm) and mouse jejunum (scan bar is 75 μm). **B** and **C**, NHE3 expression in the 3 intestinal segments in knockout (KO) and wild-type (WT) mice fed normal (N) or high (H) salt diet (n=4/group, *P<0.01 vs *Cckbr*^fl/fl^ WT, 1-way ANOVA, Holm-Sidak test). **D**, Immunofluorescence of Alexa Fluor 488-labeled F-actin (green) and Alexa Fluor 568-labeled NHE3 (red) in Dahl salt-sensitive rats (scan bar is 200 μm). **E**, Immunofluorescence of Alexa Fluor 488-labeled NHE3 (green) in WT and KO mice (scan bar is 100 μm).

Intestinal NHE3-regulated sodium absorption may participate in the regulation of BP.^14,15^ Immunofluorescence studies showed that NHE3 was expressed in intestines and colocalized with CCKBR at the microvillar brush border membranes of human intestine and mouse jejunum (Figure 3A).

NHE3 expression was increased in the duodenum and jejunum but not the ileum of *Cckbr*^fl/fl^ *villin-Cre* mice compared with *Cckbr*^fl/fl^ WT mice (Figure 3B). High-salt diet upregulated NHE3 expression in jejunum of *Cckbr*^fl/fl^ WT that was further increased in *Cckbr*^fl/fl^ *villin-Cre* mice (*P*<0.05; Figure 3C). Immunofluorescence microscopy also showed that NHE3 expression was greater in the brush border membrane of *Cckbr*^fl/fl^ *villin-Cre* mice than *Cckbr*^fl/fl^ WT mice (Figure 3D). Phospho-NHE3 in the jejunum was increased by high-salt diet, which was prevented by 2 different doses (0.02 and 0.1 mg/kg) of gastrin-SiO_2_ microspheres (Figure S12A). These results suggest a gastrin/CCKBR cross talk with NHE3 in the jejunum; gastrin-SiO_2_ microspheres treatment may mitigate high salt-induced hypertension by inhibiting NHE3 expression in the intestinal brush border membrane.

Trafficking of NHE3 in and out of the apical membrane is an important mechanism regulating its activity.^34^ Immunofluorescence study showed that NHE3 (red) is located on the surface of membrane fraction of the jejunum of the Dahl salt-sensitive rat, which was increased after a high-salt diet. Gastrin-SiO_2_ microspheres treatment markedly reduced the jejunal cell surface NHE3 expression (Figure 3D) and activity (Figure S12B) in these rats. Our study in Dahl salt-sensitive rats also showed that stool sodium (Figure S12C) in gastrin-SiO_2_ group was increased, which may the consequence of the decrease in intestinal sodium absorption. Global NHE3 knockout mice have diarrhea.^35^ There was no significant difference in stool sodium concentration of *Cckbr*^fl/fl^ *villin-Cre* and *Cckbr*^fl/fl^ WT mice fed high-salt diet (Figure S12D). The shapes and types of the feces were evaluated in gastrin-SiO_2_ microspheres group using the Bristol Stool Chart,^36^ which were normal (Bristol grade 3–4; Figure S13A). We also calculated the water in the feces (35±7%; formula in the Supplemental Material) and found no difference between *Cckbr*^fl/fl^ *villin-Cre* and *Cckbr*^fl/fl^ WT mice fed high-salt diet (Figure S13B). According to our design, gastrin-SiO_2_ microspheres do not have any ionic charge. Therefore, we measured the expression of 2 other proteins involved in intestinal ion transport. We found that high-salt diet minimally and nonsignificantly increased small intestinal Epithelial sodium channel(ENaC) expression, which was not decreased by gastrin treatment. High-salt diet increased the expression of Na+-K+-2Cl- cotransporter1(NKCC1) that was markedly decreased by gastrin-SiO_2_ microspheres treatment but not below control levels (Figure S13C through S13E). Therefore, gastrin-SiO_2_ microspheres inhibit intestinal NHE3 activity with no complicating diarrhea.

### The Mechanism of the Gastrin/CCKBR-Mediated Suppression of Intestinal NHE3 Activity

**Figure 4.**
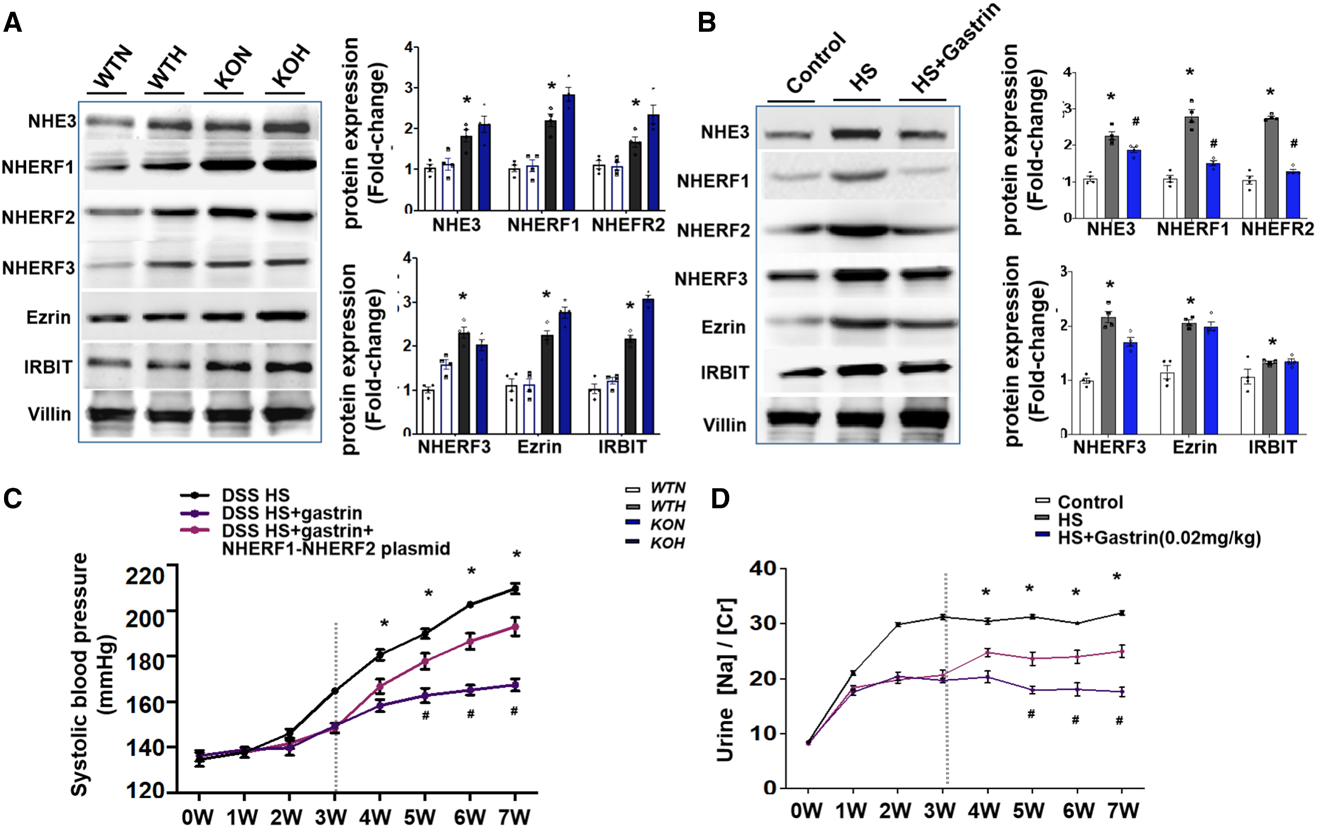
**Gastrin inhibits Na+/H+ exchanger 3 (NHE3) expression via NHERF1 and NHERF2-mediated protein trafficking. A**, Expressions of NHE3, NHERF1, NHERF2, NHERF3, ezrin, and IRBIT in intestinal brush border membranes of knockout (KO) mice and wild-type (WT) mice fed normal (N) or high (H) salt diet (n=4/group, *P<0.01 vs *Cckbr*^fl/fl^ WT, 1-way ANOVA, Holm-Sidak test). **B**, Expressions of NHE3, NHERF1, NHERF2, NHERF3, ezrin, and IRBIT in the intestinal brush border membrane of Dahl salt-sensitive (DSS) rats. Villin was used as an internal marker of intestinal epithelial cells (*P<0.05 vs DSS control [0.49% NaCl], #*P*<0.05 vs DSS + high salt [HS, 8% NaCl], 1-way ANOVA, Tukey test). **C** and **D**, AAV-NHERF1 and AAV-NHERF2 plasmids impair the effectiveness of the gastrin-SiO_2_ microspheres in ameliorating the increase in systolic blood pressure (BP) and impairing the increase in urine Na concentration in DSS rats fed HS diet (*P<0.05 vs others, # vs DSS + others, 1-way ANOVA, Tukey test).

Electroneutral NaCl transport requires the formation of macromolecular complexes, including that caused by NHE3, which is mediated by the NHE regulatory factor (NHERF) family of scaffold proteins.^37,38^ Ezrin and NHERFs interact with NHE3,^39^ and inositol-1,4,5-trisphosphate (IP3) receptor-binding protein (IRBIT) is an NHE3-binding partner, involving the phospholipase C (PLC)/PKC (protein kinase C) pathway.^40^ The expressions of NHE3, NHERF1, NHERF2, NHERF3, ezrin, and IRBIT were higher in *Cckbr*^fl/fl^ *villin-Cre* mice than *Cckbr*^fl/fl^ WT mice (Figure 4A), which were further increased by high-salt diet. Gastrin-SiO_2_ microspheres downregulated high salt elevated expressions of NHE3, NHERF1, and NHERF2 but not NHERF3, ezrin, or IRBIT in Dahl salt-sensitive rats (Figure 4B). The intravenous injection of the combination of Adeno-associated virus(AAV)-NHERF1 and AAV-NHERF2 plasmids partially counteracted the gastrin-SiO_2_ microspheres-induced amelioration of the high BP and urine Na concentration in Dahl salt-sensitive rats (Figure 4C and 4D).

**Figure 5.**
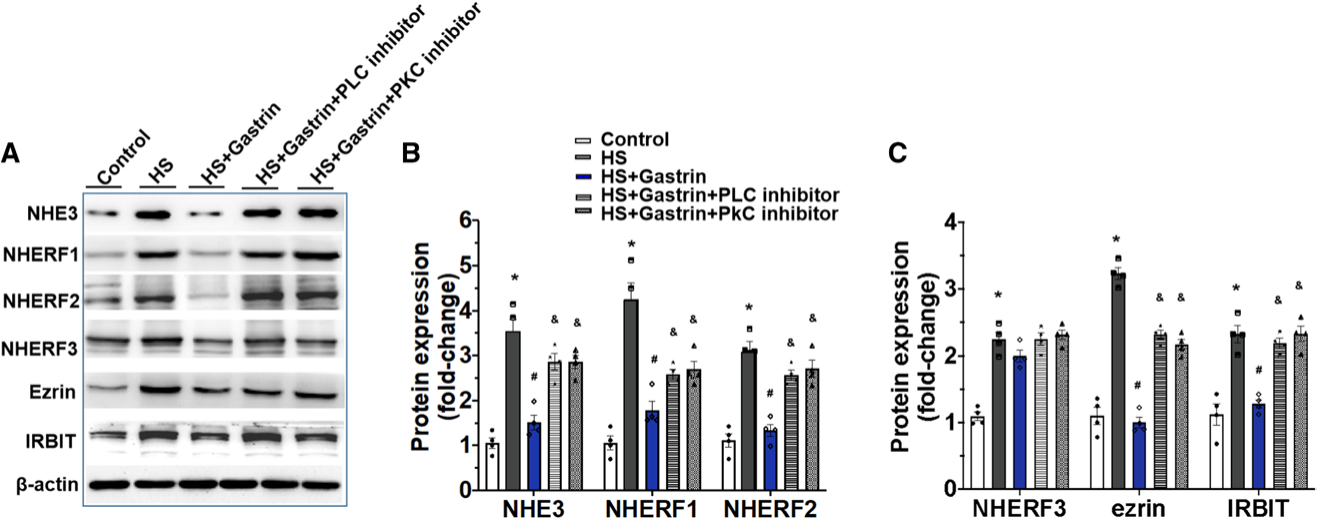
**Gastrin-mediated Na+/H+ exchanger 3 (NHE3) inhibition via a phospholipase C (PLC)/PKC (protein kinase C)-dependent manner. A–C**, Protein expressions of NHE3, NHERF1, NHERF2, NHERF3, Ezrin, and IRBIT on membranes of Caco-2 cells treated with 140 mmol/L Na^+^ (control), 240 mmol/L Na^+^ (high salt [HS]), HS incubated cells pretreated with gastrin (10^−8^ mol/L, HS + gastrin), U73122 (PLC inhibitor, 5×10^−6^ mol/L) + gastrin, or Go6983 (PKC inhibitor, 5×10^−6^ mol/L) + gastrin (*P<0.05 vs control, # vs HS, & vs HS + gastrin, 1-way ANOVA, Tukey test). KOH indicates knockout mice on high-salt diet; KON, knockout mice on normal-salt diet; WTH, WT mice on high-salt diet; and WTN, control group of WT mice on normal-salt diet.

**Figure 6.**
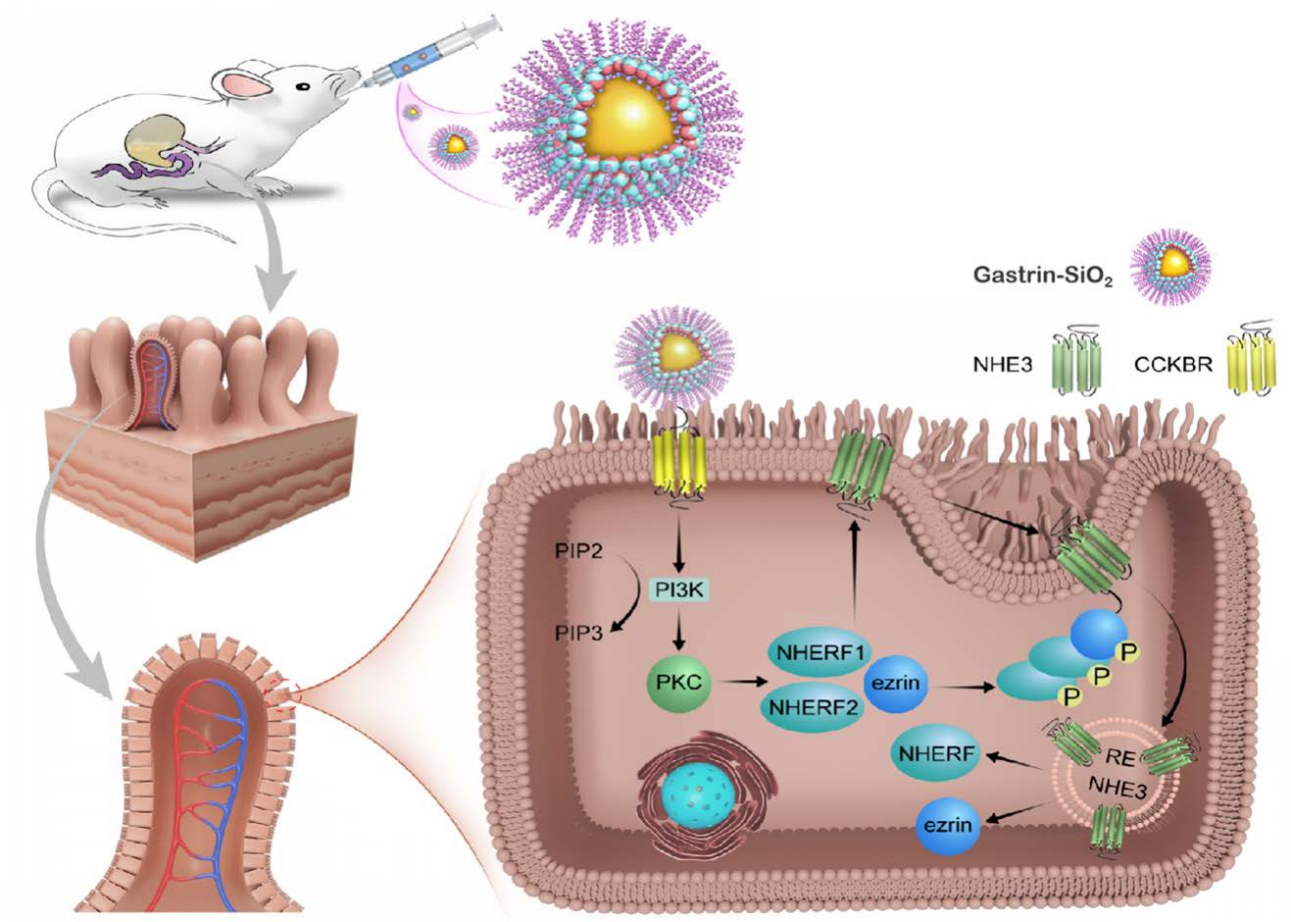
Gastrin-SiO_2_ microspheres effectively inhibit Na+/H+ exchanger 3 (NHE3) activity caused by NHERF1 and NHERF2-inducedvsmall intestinal brush border membrane NHE3 trafficking, through a phospholipase C (PLC)/PKC (protein kinase C)-dependent pathway.

High-salt concentration increased NHE3, NHERF1, NHERF2, NHERF3, ezrin, and IRBIT expressions, which were decreased by gastrin in Caco-2 cells. A PLC or PKC inhibitor blocked the gastrin-mediated decrease in these protein (Figure 5A through 5C). These results demonstrated that the gastrin/CCKBR-mediated amelioration of salt-sensitive hypertension-related genes, and thus hypertension, is via a reduction of NHERF1-NHERF2-induced small intestinal brush border membrane NHE3 trafficking, through a PLC/PKC-dependent pathway (Figure 6).

## Discussion

Gastrin, by stimulating its receptor, CCKBR,^26^ exerts a natriuretic effect due to inhibition of NHE activity, such as NHE3.^25,41^ Gastrin can also induce the expression of its own receptor (*CCKBR*), which would further amplify cellular CCKBR signaling.^42^ Genome-wide association studies have shown that the chromosomal loci of *CCKBR* (11p15.5)^43^ and *GAST* (17q21)^44^ are linked to human essential hypertension.^45^ Germline deletion of *Gast*^46^ or *Cckbr*^27^ in mice decreases urine sodium concentration and increases BP. Pentagastrin can also decrease intestinal sodium absorption that is potentiated by stimulation of cholinergic or inhibition of sympathetic nerves.^47^ To determine if intestinal CCKBR, independent of renal CCKBR, can regulate BP by inhibiting intestinal NHE3, we generated mice lacking *Cckbr* only in the intestines, that is, *Cckbr*^fl/fl^ *villin-Cre* mice. *Cckbr*^fl/fl^ *villin-Cre* mice had increased BP and elevated duodenal and jejunal NHE3 expression. The expression of NHE3 is highest in the jejunum, followed by the duodenum, and lowest in the ileum^48^; NHE3 is also expressed in the colon.^49^ However, only about 4% of fluids and electrolytes are absorbed in the colon.^50^ Therefore, lack of CCKBR in the intestines can increase sodium balance by stimulating intestinal sodium absorption, via NHE3.

The gavage of gastrin-SiO_2_ microspheres mitigated the high-salt diet-induced hypertension in Dahl salt-sensitive rats with a decrease in intestinal NHE3 expression and activity, increase in stool sodium. Inhibition of NHE3 activity in the gut decreases BP but causes heavy diarrhea.^15,17^ In our study, the stool shapes and water content were normal, which effectively prevented the side effect of diarrhea. In addition to NHE3, ENaC-mediated electrogenic Na absorption is important for fluid and electrolyte absorption in the distal colon.^51^ Decreased ENaC activity is involved in the pathogenesis of diarrhea.^52^ In our study, ENaC expression was not affected by gastrin-SiO_2_ microspheres treatment. The rate of epithelial Cl secretion is determined, in large part by the activity of basolateral transporters, such as NKCC1.^51^ The intestinal expression of NKCC1 was increased by high-salt diet and markedly decreased by gastrin-SiO_2_ treatment but not below control levels. Therefore, normal expression of ENaC and NKCC1 after gastrin-SiO_2_ microspheres treatment may have compensated for the inhibition of NHE3, thus preventing a marked increase in stool sodium and, therefore, the development of diarrhea.

There are beneficial effects of inhibition of intestinal NHE3 in addition to BP regulation and end-organ protection.^28,54^ The long-term subcutaneous infusion of gastrin for 7 to 28 days protected against hypertensive nephropathy by normalizing BP, decreasing renal tubule cell apoptosis, and increasing macrophage efferocytosis^55^ Gastrin treatment for 28 days also exerted a protective effect on myocardial infarction.^56^ In our study, the high-salt diet-mediated increase in renal injury was mitigated by gastrin-SiO_2_ microspheres treatment for 7 weeks.

Gastrin acts as a growth factor for the gastric oxyntic mucosa and plays a role in carcinogenesis, colorectal neoplasia, in particular.^57,58^ The carcinogenic properties of gastrin has been mainly described with increased circulating gastrin.^29^ As aforementioned, ingestion of gastrin-SiO_2_ does not cause hypergastrinemia. We also found that carcinogenic and inflammatory factors in the small intestines were not increased by gastrin-SiO_2_ microspheres. The gut microbiome participate in the development of hypertension.^59–61^ Excessive salt intake leads to changes in intestinal microbiota and promotes the activation of innate and adaptive immune systems, resulting in salt-sensitive hypertension.^62^ However, only few studies have explored on the role of gastrin in the gut microbiome. One study showed that charred *Crataegi fructus* can promote gastrin secretion and restore the composition of disturbed intestinal microbiota to normal levels, including *Bacteroides*, *Akkermansia*, and *Intestinimonas*.^63^ Therefore, the beneficial effect of orally administered gastrin-SiO_2_ microspheres on gut microbiota could be the subject of future studies.

In a population study of working-class Brazilians, hypertension increased the risk nonalcoholic fatty liver disease. Hypertension is an independent of predictor of advanced liver; other predictors were high serum ALT and C-peptide levels.^64^ High-salt diet aggravated the BP of spontaneously hypertensive rats was associated with increased serum ALT levels.^65^ Dahl salt-sensitive rats fed a high-salt diet are hypertensive and hyperlipidemic.^66^ We also found that a high-salt diet increased serum ALT but not AST levels in *Cckbr^fl/fl^* WT mice. By contrast, high-salt diet increased both serum ALT and AST levels in *Cckbr^fl/fl^ villin-Cre* mice and Dahl salt-sensitive rats that were normalized by gastrin-SiO_2_ microspheres treatment. But, the food intake (g/wk) and body weight (g) were similar among the these groups. Therefore, the elevated serum ALT and AST levels is not caused by excessive food intake.

We suggest a unifying mechanism that may account for the decrease in intestinal NHE3 expression induced by gastrin/CCKBR stimulation. This involves a decrease in NHERF1 and NHERF2 expression and stimulation of the PI3K (phosphatidylinositol 3-kinase)/PKC pathway. Among the NHERFs, NHERF1, NHERF2, and NHERF3, have the highest tendency to complex with and inhibit NHE3 activity.^57,68^ In NHERF1-deficient mice, the intestinal brush border expression of NHE3 is normal but total NHE3 expression is reduced.^69^ By contrast, in NHERF2-null mice, basal NHE3 activity is decreased, associated with decreased expression of NHE3 in the apical membrane, without a change in total NHE3 expression.^70^ We found that intestinal NHERF1 and NHERF2 overexpression minimized the gastrin-SiO_2_ microspheres-induced amelioration of BP and decrease in urinary sodium, reinforcing the importance of NHERF1 and NHERF2 in the stimulation of NHE3 activity. The activation of p38 MAP (mitogen-activated protein) kinase, followed by the activation of MAPK (MAP kinase activated kinase 2)/APK-2, PI3K, and Akt2, leads to brush border membrane NHE3 translocation and stimulation of NHE3 activity.^71^ Our in vitro study in Caco-2 cells indicated that NHERF1 and NHERF2, but not NHERF3, ezrin, and IRBIT participate in the gastrin-mediated NHE3 inhibition via a PLC/PKC-dependent manner. We suspect that there may be other signaling pathways involved in the regulation of NHE3 translocation, which need further exploration. We conclude that intestinal gastrin-SiO_2_ microspheres treatment inhibits NHE3 activity by reducing cell surface NHE3 protein through a NHERF1-NHERF2 and PLC/PKC pathway.

## Perspectives

By using in vivo and in vitro experiments, we demonstrated that gastrin-SiO_2_ microspheres, via intestinal CCKBR, ameliorated salt-sensitive hypertension and organ damage by partial inhibition of NHE3 activity that is PLC/PKC dependent, without causing diarrhea. Gastrin-SiO2 microspheres, which cannot be absorbed into the circulation, reduce the risk of inflammation and cancer. These observations broaden our understanding of the function of intestinal gastrin/CCKBR and mechanisms of intestinal sodium transport. Our study indicates that targeting intestinal CCKBR is a prospective clinical therapeutic strategy for salt-sensitive hypertension, by inhibiting inappropriate intestinal sodium absorption.

### Additional Information

Data on additional characterizations of the reagents, such as FITC fluorescent and FT-IR spectra of SiO_2_ and SiO_2_-NH_2_, analysis of gastrin-SiO_2_ microspheres and detection of serum gastrin by mass spectrometry, measurement of serum markers of colon cancer and intestinal inflammation, H&E staining and analysis of the biocompatibility of gastrin-SiO_2_ microspheres are shown in the Supplemental Material.

## Article Information

### Sources of Funding

These studies were supported, in part, by grants from the Chinese Academy of Medical Sciences Innovation Fund for Medical Sciences (CIFMS, CAMS-I2M, 2021-I2M-1-072), the National Natural Science Foundation (China; 81970358, 81800402, and 82100902), Beijing Outstanding Young Scientist Program (grant no. BJJWZYJH 01201910010024), and the National Institutes of Health (United States; (R01DK039308, P01HL074940, R01DK119652, P.A. Jose).

### Disclosures

None.

## Supplementary Material


