# Supplementary material for: Intestinal Gastrin/CCKBR (Cholecystokinin B Receptor) Ameliorates Salt-Sensitive Hypertension by Inhibiting Intestinal Na+/H+ Exchanger 3 Activity Through a PKC (Protein Kinase C)-Mediated NHERF1 and NHERF2 Pathway
Source: Hypertension. 2022 Jun 8;79(8):1668–79. doi: 10.1161/HYPERTENSIONAHA.121.18791 (PMC9278716; doi:10.1161/HYPERTENSIONAHA.121.18791)
Supplement: Supplementary file 1 [file hyp-79-1668-s001.pdf]

Intestinal Gastrin/CCKBR ameliorates salt-sensitive hypertension by inhibiting intestinal Na^+/H^+ exchanger 3 activity through a PKC-mediated NHERF1 and NHERF2 pathway

Authors

Xiaoliang Jiang[1,†], Yunpeng Liu[1,†], Xin-Yang Zhang[2,†], Xue Liu[1], Xing Liu[1],

Xianxian Wu[1], Pedro A Jose[3,4], Shun Duan[2*], Fu-Jian Xu[2*], Zhiwei Yang[1*]

Affiliations

[1]NHC Key Laboratory of Human Disease Comparative Medicine (The Institute of Laboratory Animal Sciences, CAMS&PUMC), National Human Diseases Animal Model Resource Center, Beijing Engineering Research Center for Experimental Animal Models of Human Critical Diseases. 5 Pan Jia Yuan Nan Li, Chaoyang District, Beijing 100021, P.R. China.

[2]Key Lab of Biomedical Materials of Natural Macromolecules (Beijing University of Chemical Technology), Ministry of Education, Beijing Laboratory of Biomedical Materials, Beijing Advanced Innovation Center for Soft Matter Science and Engineering, Beijing University of Chemical Technology, Beijing 100029, P.R. China.

[3]Department of Pharmacology and Physiology, The George Washington University School of Medicine & Health Sciences, Washington, DC, 20052, USA.

[4]Department of Medicine, Division of Kidney Diseases & Hypertension, The George Washington University School of Medicine & Health Sciences, Washington, DC, 20052, USA.

†These authors contributed equally to this work.

Short Title: Intestine Gastrin/CCKBR and salt-sensitive hypertension

* Corresponding author
* Zhiwei Yang, MD, PhD
Professor of Physiology
Institute of Laboratory Animal Science, Chinese Academy of Medical Sciences (CAMS) & Peking Union Medical College (PUMC).
5 Pan Jia Yuan Nan Li Chaoyang District, Beijing 100021, P.R. China
Tel: 0086-10-67776809 Fax: 0086-10-67770685
Email: yangzhiwei@cnilas.pumc.edu.cn

Supplemental File

Methods

Studies in *Cckbr*$^{fl/fl}$ v*illin-Cre* mice

Experiments were performed in 6- to 8-week-old male C57BL/6J mice (Huafukang), *Cckbr*$^{fl/fl}$ WT mice, and *Cckbr*$^{fl/fl}$ *villin-Cre* mice (VIEWSOLID BIOTECH). In this study, *Cckbr*$^{fl/fl}$ mice and *Cckbr*$^{fl/fl}$ *villin-Cre* mice were fed normal salt (0.49% NaCl) and high salt (4% NaCl) diets. The BPs of the mice were measured as previously described[23]. The intestines were collected for determination of NHE3 and NHE3-related complex protein expressions. All animal protocols were approved by the Institutional Animal Care and Use Committee of the Institute of Laboratory Animal Science[YZW2019002].

Gastrin-SiO_2 microspheres absorption study in rats

Gastrin-SiO_2 microspheres absorption in the intestines was determined by performing serum mass spectrometry (**Figure S1**). Male Dahl salt-sensitive rats (6 to 8 weeks old, 180 to 200 g; Huafukang), rats were randomized into 3 groups (five rats per group). The rats in the first group were fed normal salt (0.49% NaCl) diet whereas the rats in the second group were fed high salt (8% NaCl) diet. The rats in the third group were fed high salt diet and gavaged daily with Gastrin-SiO_2 microspheres in water for 6 weeks. We performed a dose-response study **(Figure S2)** and used in the current experiments the lower dose of Gastrin-SiO_2 (0.02 mg/kg), with a calculated gastrin concentration of 2597 pM, which is in the range of a published study[31] . BP was measured 3 times a week by radiotelemetry [23]. The weekly BPs were the average of three measurements per week at 8:30 AM. Urine and stools were collected every week in rats individually kept in metabolic cages. After 7 weeks, the rats were anesthetized with 2% isoflurane and sacrificed by an overdose of pentobarbital after blood, kidneys, and intestines were collected.

Study design

The study was designed to elucidate the mechanism(s) by which gastrin lowers BP or keeps BP in the normal range through the inhibition of NHE3 expression and activity in the small intestines. CCKBR was specifically and selectively stimulated in the intestines by using gastrin-bound to SiO_2 in microspheres (Gastrin-SiO_2), prepared by the Beijing University of Chemical Technology. We evaluated the effects of gastrin delivered by Gastrin-SiO_2 microspheres on the expression and activity of intestinal NHE3, renal function, including urine electrolytes, fecal sodium, and BP in Dahl salt-sensitive rats and mice with intestine-specific deletion of the gastrin receptor, *Cckbr*$^{fl/fl}$ *villin-Cre*. The rodents were randomized into wild-type control, vehicle-treated control, and *Cckbr*$^{fl/fl}$ *villin-Cre* groups.

Chemical tools

Gastrin(p-Glu-Gly-Pro-Trp-Leu-Glu-Glu-Glu-Glu-Glu-Ala-Tyr-Gly-Trp-Met-Asp-Phe-NH2) (prepared by Beijing University of Chemical Technology), NHE3

(27190-1-AP, ProteinTech), NHERF1 (ab3452, Abcam), NHERF2 (YT5676, Immunoway), NHERF3 (ab121248, Abcam), ezrin (ab4069, Abcam), IRBIT (ab178693, Abcam), villin (ab109516, Abcam), CCKBR (ab77077, Abcam), F-actin (ab205, Abcam), MMP2 (10373-2-AP, ProteinTech), MMP9 (A2095, Abclone), Go 6983 (PKC inhibitor) (2285, Tocris), and U73122 (PLC inhibitor) (1268, Tocris) were used.

Cell culture and high salt treatment

Human Caco-2 cells were purchased from Cell resource center of Institute of Basic Medical Sciences, Peking Union Medical College, Chinese Academy of Medical Sciences. The cells were cultured in MEM-EBSS medium containing 10% FBS, 1% penicillin, 1% streptomycin, 1% NEAA, and 10 mM Hepes at 37ºC in 5% CO_2. The cells were cultured for 12 days after becoming confluent. The cells were divided into five groups: vehicle group (135 mmol/L Na^+), high salt group (185 mmol/L Na^+), high salt + gastrin group(10^{-8} mol/L), gastrin + PLC inhibitor (Go6983), and gastrin + PKC inhibitor (U73122). The PLC- and PKC-inhibited groups were pretreated with Go6983 (5 x 10^{-6} mol/L) and U73122 (5 x 10^{-6} mol/L), respectively for 30 min. The vehicle and gastrin groups were also incubated for 30 min prior to treatment. Then, vehicle was added into the vehicle and high salt groups and gastrin in the gastrin, PKC-, and PLC-inhibited groups and incubated for another 30 min. The Caco-2 cells were cultured for 24 h prior to vehicle or drug treatment.

Preparation of brush border membranes (BBMs) and immunoblotting

The small intestines were rinsed with ice-cold 0.9% saline and opened along the mesenteric border. The mucosa was scraped with a glass slide, and then transferred in 15 ml cold buffer I (300 mM D-mannitol, 5 mM EGTA, 12 mM Tris-base, pH 7.1) with protease inhibitors (Complete Protease Inhibitor Cocktail). Following a 2 min homogenization, 20 mL cold water and 12 mM $MgCl_2$ were added and mixed by inversion. The samples were centrifuged at 2000 g at 4 °C for 15 min to remove cell debris and aggregated membranes. The supernatants were centrifuged at 30000 g at 4°C for 30 min. The pellets were resuspended in buffer II (150 mM D-mannitol, 2.5 mM EGTA, 6 mM Tris-HCl, pH 7.1), precipitated with 12 mM $MgCl_2$ on ice for 15 min, and centrifuged at 30000 g at 4 °C for 30 min. The final BBM pellets were resuspended in PBS and stored at -80 °C. The tissue extracts were homogenized in ice-cold RIPA buffer with protease inhibitors. The homogenates were placed on ice for 30 min and then centrifuged at 10000 g for 30 min. The supernatants were collected and stored at -80 °C. The protein concentrations were quantified using BCA kits (Beyotime Biotechnology). Western blotting was conducted for CCKBR, NHE3, NHERF1, NHERF2, NHERF3, ezrin, IRBIT, MMP2, and MMP9.

Histological analyses

The intestines were fixed in 4% paraformaldehyde and embedded in paraffin. Four μm-thick sections were obtained (Leica RM2235; Leica Microsystems and Masson trichrome stained (Solarbio, Beijing, China) to identify collagen deposition. Masson trichrome stains were scanned using Nano Zoomer S60 (Hamamatsu).

Immunofluorescence

The intestines were prepared as above except that immunofluorescent primary antibodies were used: CCKBR (ab77077, Abcam), NHE3 (27190-1-AP, ProteinTech), Villin (ab109516, Abcam), and F-actin (ab205, Abcam); DAPI was used to stain the nucleus.

Quantitative real-time polymerase chain reaction (PCR):

Total RNA was purified using the RNeasy RNA Extraction Mini kit (Qiagen, Valencia, CA, USA). The RNA samples were converted into first strand cDNA, using an RT2 First Strand kit, following the manufacturer's protocol (Qiagen). Quantitative gene expression was measured by real-time qPCR, performed on an ABI Prism 7900 HT (Applied Biosystems, Foster City, CA, USA). The assay used gene specific primers (Qiagen) and SYBR Green real time PCR detection method. (Qiagen) was used as described in the manufacturer's manual. The primers used are Forward primer 5'-ATGGGCTCCCTCTCATCAGT-3', Reverse primer 5'-GCTTGGTGGTTTGCTACGAC-3' (TNFα); Forward primer 5'-TAGTCCTTCCTACCCCAACTTCC-3', Reverse primer 5'-TTGGTCCTTAGCCACTCCTTC-3' (IL-6); Forward primer 5'-ATGCAGGTCTCTGTCACGCTTCTGGGC-3', Reverse primer 5'-CTAGTTCTCTGTCATACTGGTCAC-3' (MCP-1); Forward primer 5'-CCCAGCCCTATGCCTTTTCA-3', Reverse primer 5'-ACAGGTGCAGACAGGGATTG-3' (NFκB); Forward primer 5'-ATGATTCTACCCACGGCAAG-3', Reverse primer 5'-CTGGAAGATGGTGATGGGTT-3' (GAPDH); The expressions of TNFα, MCP-2, MCP-1, NFκB, IL-6, and IL-1β were normalized to those of GAPDH and analyzed using the 2 ΔΔCt method.

Measurement of NHE3 activity

Cellular NHE3 activity was measured in $CaCo_2$ cells, using the intracellular pH-sensitive dye BCECF-AM. The cells were serum-starved for 4 h before measuring NHE3 activity. HOE-642 (10 μM) was included to inhibit the contribution of NHE1. The cells were loaded for 20 min at 37°C with 10 μM BCECF-AM in 50 mM Na^+/NH_4Cl solution (98 mM NaCl, 5 mM KCl, 2 mM $CaCl_2$, 1 mM $MgSO_4$, 1 mM NaH2PO4, 25 mM glucose, 20 mM HEPES, and 40 mM NH_4Cl, pH 7.4). The cells were alternately excited at 440 and 495 nm and the fluorescence emission at 530 nm. The 495/440 excitation ratio corresponds to a specific pHi. The BCECF signal was calibrated for 15 min using a high K^+ solution (20 mM NaCl, 130 mM KCl, 1 mM $MgCl_2$, 1 mM $CaCl_2$, 5 mM HEPES) containing 10 μM nigericin.

Serum biochemistry

Routine serum biochemical parameters, including alanine aminotransferase (ALT), aspartate aminotransferase (AST), low density lipoprotein-cholesterol (LDL-C), creatine kinase (CK), urea nitrogen (UN), and uric acid (UA) were measured by Mindray BS-360 automatic biochemical analyzer and related reagents (Mindray, China).

ELISA

Commercially available ELISA kits for rat were used, according to the manufacturer's instructions. CA19-9 (LS-F27918, LSBio), PSA (E-EL-R0796c, Elabscience), CEA (ZY-CEA-Ra,

ImmunoClone).

Stool form and water content

The shapes and types of the feces in each group is elevated by the Bristol Stool Chart, grade 1-2 (dry and hard) ;3-4 (normal) ;5-7 (diarrhea).which is normal (Bristol grade 3-4). The water of feces is calculated by the formula:

$$\text{feces water} = \frac{(\text{fecal weight before drying} - \text{fecal weight after drying})}{\text{feces weight before drying}} \times 100.$$

Fluorescence emission computed tomography (FLECT)

FLECT was performed using the Trifoil InSyTe FLECT® imager (beta version, TriFoil Imaging, USA). The rats were gavaged with Gastrin-SiO_2-FITC; 24 h later the rats were anesthetized with isoflurane (4% for induction, 1.5% for maintenance in 30% oxygen and 70% nitrous oxide), maintenance in 30% oxygen and 70% nitrous oxide), to obtain a three-dimensional image, using 488 nm excitation laser and 525 nm filter. The absorption data were collected from 29 slices with 1 mm spacing and 29 source angles per slice. Reconstruction of the FLECT image was performed using 1 mm 3 grid with attenuation correction and 1,000 iterations to generate the reconstructed three-dimensional image of each scan. Following the FLECT scan, a computed tomography (CT) scan was performed in each mouse using X-ray voltage of 45 kVp, exposure time of 1,500 ms, and over 360° projections to generate FLECT images, which were overlaid onto its respective FLECT image. FLECT and CT scan image files were then coregistered, fused, analyzed, and quantified using the analysis software In Vivo Scope version 2.00.

Lentivirus production.

The Lentivirus HBLV-r-Slc9a3r1-3*flag-GFP and HBLV-r-Slc9a3r2-3*flag-GFP 6*10^8 TU/mL was constructed in HanBio Biotechnology(Shanghai, China), as follows. The transfer vectors pHBLV encoding enhanced flag and luciferase (GFP). To clone the pcDNA Slc9a3r1 and Slc9a3r2 into the Lentivirus vector, PCR was conducted using the following primers: 5′-GAGGATCTATTTCCGGTGAATTCGCCACCATGGCCTCCACCTTCAAC -3′ and 5′-TAGTCACTTAAGCTTGGTACCGAGGATCCCATCACCGTGTCTTCAGAG-3′. The PCR product was digested with Cla I and BamH I, and then inserted into a Lentivirus vector with an expression cassette consisting of a CMV promoter to construct NHERF1 and NHERF2 overexpression lentiviruses. Vector was resuspended in complete Dulbecco's modified Eagle's medium and stored at -80°C. Total unit (TU)/ml was determined for each vector stock by counting GFP-positive cells in diluted 293T cells. All preparations of lentivirus underwent quality control for presence of replication-competent lentivirus by monitoring p24 antigen expression. Lentivirus vector stocks with titers ranging from 0.1 to 6 × 109 TU/ml were used.

Statistical analyses

Statistical difference between 2 groups was determined using a 2-tail Student’s t test. Multiple group comparisons were performed using one-way ANOVA, followed by Dunnett’s post-hoc test. All statistical analyses and determination of statistical significance ($p \leq 0.05$) were performed using SPSS 19.0 software (IBM SPSS Statistics). The graphs were generated by Prism 6 software (GraphPad Software

Inc.). Data are expressed as mean ± SEM.

Supplemental Tables

	Control	HS	HS+Gastrin	P value (NS vs HS)	P value (HS vs HS+Gastriin)
The first week	217.53±4.92	220.87±3.52	215.71±2.25	0.65	0.61
The second week	245.36±2.87	246.31±2.72	247.48±4.65	0.34	0.29
The third week	261.33±4.50	260.60±5.62	264.29±4.13	0.36	0.18
The fourth week	273.14±5.30	271.75±4.32	269.35±3.79	0.51	0.09
The fifth week	289.32±3.10	291.75±7.83	293.75±3.47	0.25	0.44
The sixth week	298.43±6.30	300.16±4.82	302.42±5.90	0.61	0.61
The seventh week	317.73±4.40	318.23±7.39	320.50±5.53	0.12	0.10
The eighth week	325.97±3.91	327.07±8.47	329.89±1.03	0.23	0.70

Table S1 Body weights (g) of Dahl salt-sensitive rats fed normal salt diet (Control), high salt (HS), or HS and Gastrin-SiO_2 microspheres (HS+Gastrin).

	Control	HS	HS+Gastrin	P value (NS vs HS)	P value (HS vs HS+Gastriin)
The first week	103.71±5.46	107.98±6.14	109.24±5.56	0.77	0.39
The second week	109.43±1.45	114.12±2.72	108.63±1.48	0.55	0.47
The third week	113.53±2.32	116.24±6.42	116.89±7.13	0.12	0.99
The fourth week	126.44±1.36	124.69±3.69	121.32±3.11	0.41	0.29
The fifth week	127.51±2.31	127.98±0.83	129.35±3.47	0.26	0.68
The sixth week	129.66±1.33	129.01±5.12	130.93±6.40	0.51	0.21
The seventh week	131.47±2.49	133.76±1.98	134.04±2.34	0.14	0.37
The eighth week	129.54±4.63	133.87±4.21	128.65±3.98	0.11	0.17

Table S2 Food intakes (g/week) of Dahl salt-sensitive rats fed normal salt diet (Control), high salt (HS), or HS and Gastrin-SiO_2 microspheres (HS+Gastrin).

Supplemental Figures

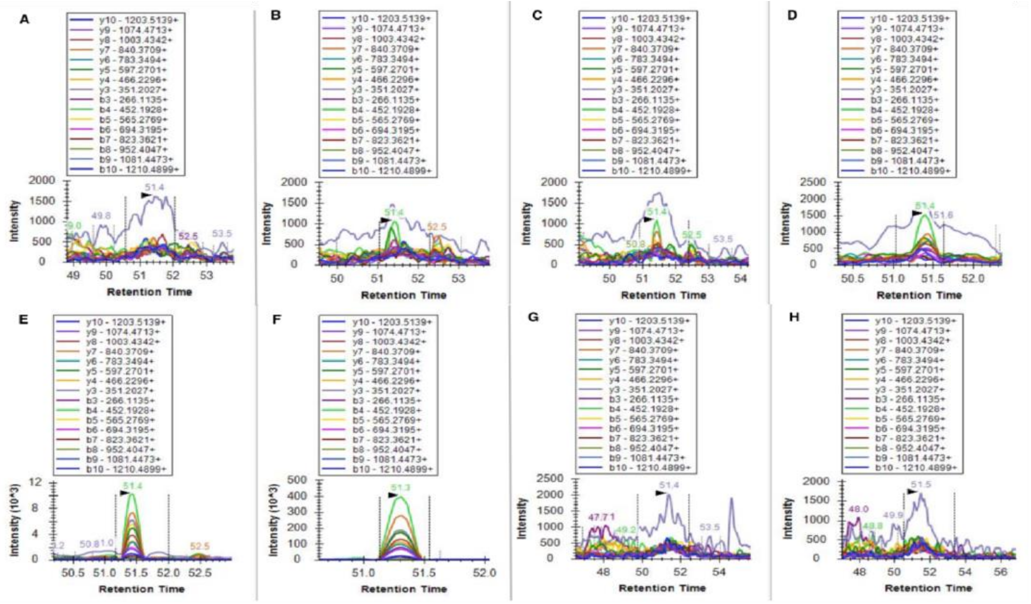

Figure S1 Serum gastrin detection in Control and Gastrin-SiO_2 microspheres-gavaged Dahl salt-sensitive rats. The sera of Dahl salt-sensitive rats were tested for the presence of synthetic gastrin by mass spectrometry. A-F: the signal response plots for different concentrations of standard peptides, A: 200 pg/µl, B: 500 pg/µl, C: 1 ng/µl, D: 2 ng/µl, E: 5 ng/µl, F:10 ng/µl. G: serum signal response plots of control group. H: serum signal response plots of Gastrin-SiO_2 microspheres-gavaged group.

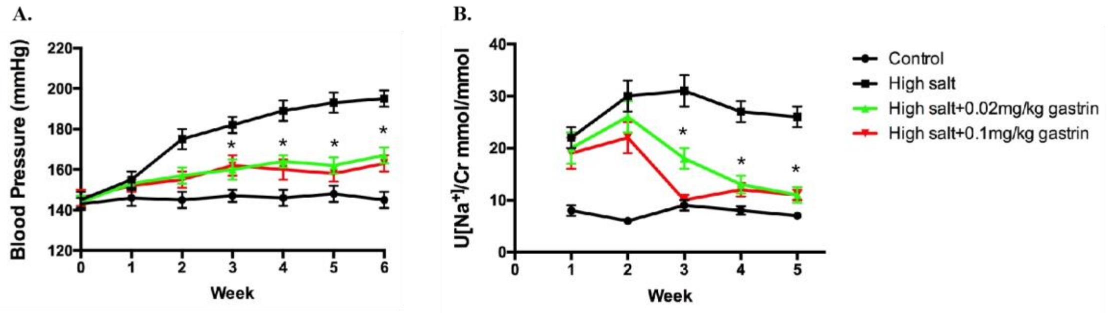

Figure S2. (A) Systolic BP was measured from the femoral artery by telemetry and the data were analyzed by Acqknowledge 5.0 software. **(B)** Urinary sodium was measured by flame photometry（*P <0.05 vs High salt group, one-way ANOVA, Tukey test, n=4/group).

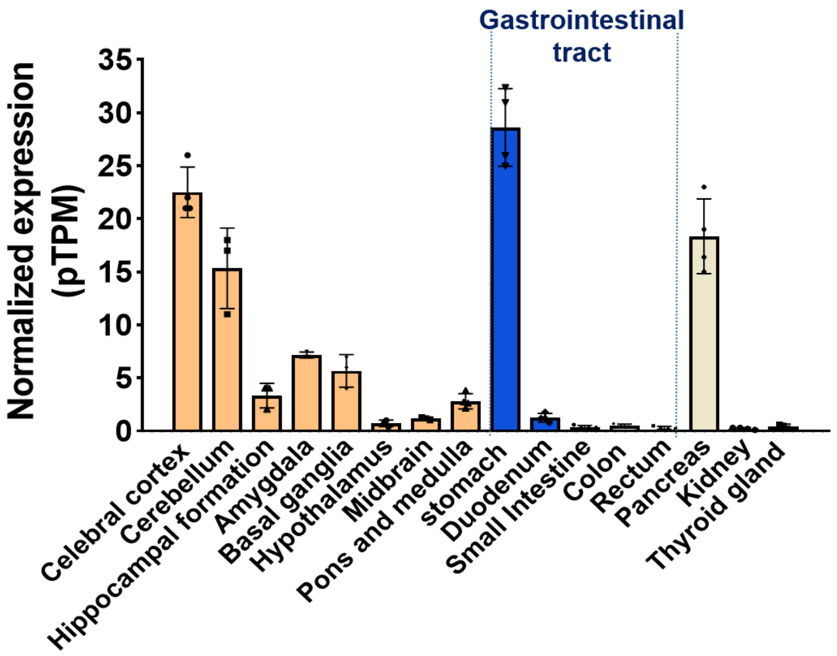

Figure. S3 The data from HPA, GETx, and FANTOM5 databases are inputted into GraphPad Prism 5 to graph human *CCKBR* mRNA organ expression.

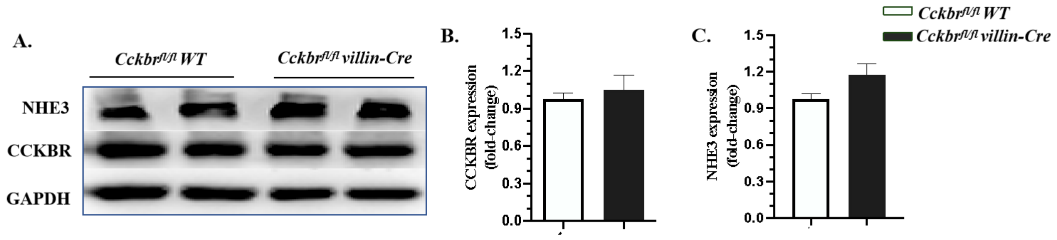

Figure. S4 (A-C) NHE3 and CCKBR protein expression in the kidney of *Cckbr*$^{fl/fl}$ wild-type (WT) *Cckbr*$^{fl/fl}$ *villlin-Cre* (knockout mice), quantified by western blot (n=4/group).

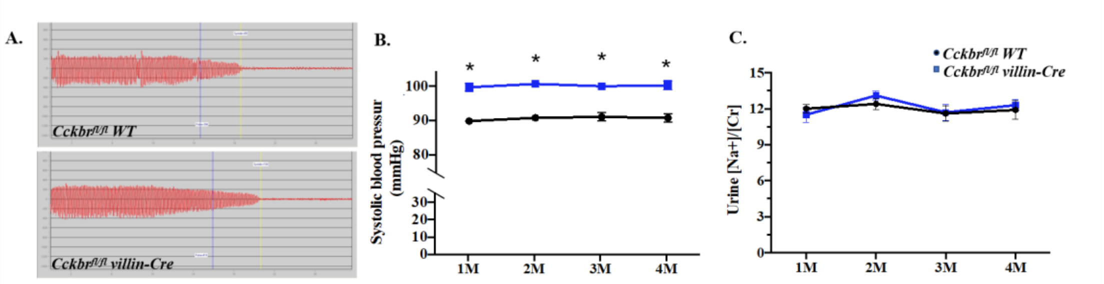

Figure S5 (A) Schematic diagram of blood pressure fluctuations. **(B)** Systolic blood pressure was measured by telemetry and the data were analyzed by Acqknowledge 5.0 software. **(C)**

Urine sodium was measured by flame photometry（*P <0.05 vs *Cckbr*$^{fl/fl}$ *WT*, one-way ANOVA, Tukey test).

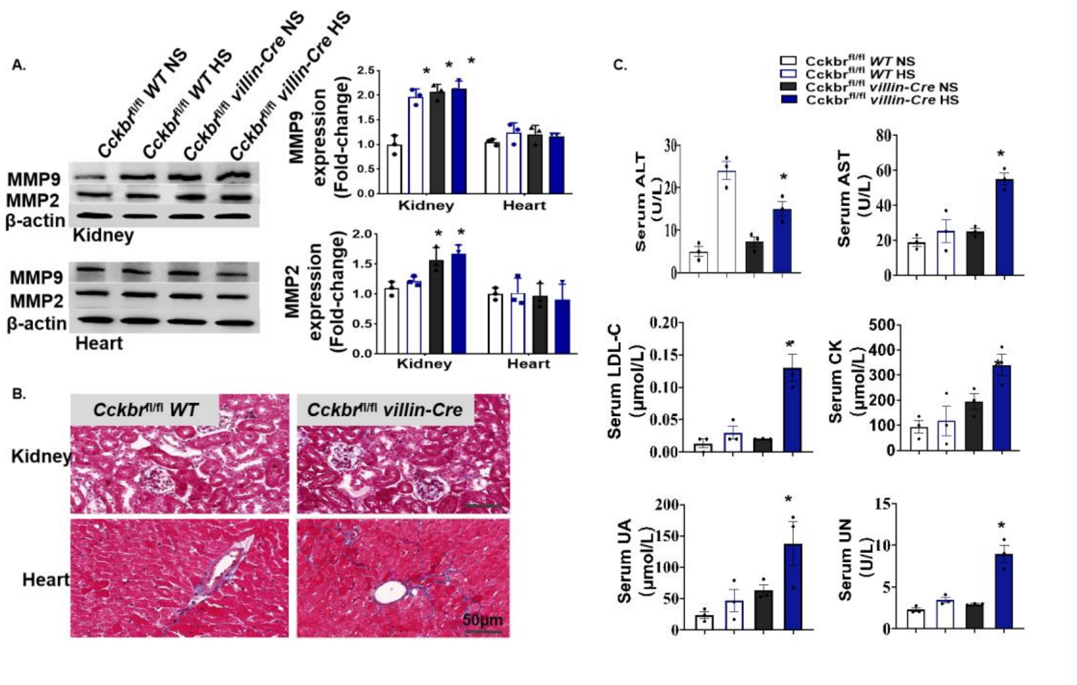

Figure S6 (A) MMP9 and MMP2 protein expression quantified by Western blot (n=4/group, *P<0.01 vs *Cckbr*$^{fl/fl}$ WT+NS, one-way ANOVA, Holm-Sidak test).**(B)** Masson-stained sections of the kidney and heart (scan bar is 50 μm) of *Cckbrfl/fl* WT mice and *Cckbrfl/fl villin-Cre* (KO) mice. **(C)** Serum biochemical measurements of alanine transaminase (ALT), aspartate transaminase (AST), low density lipoprotein-cholesterol, LDL-C, creatine kinase (CK), uric acid (UA), and urea nitrogen (UN). NS=normal salt, HS=high salt

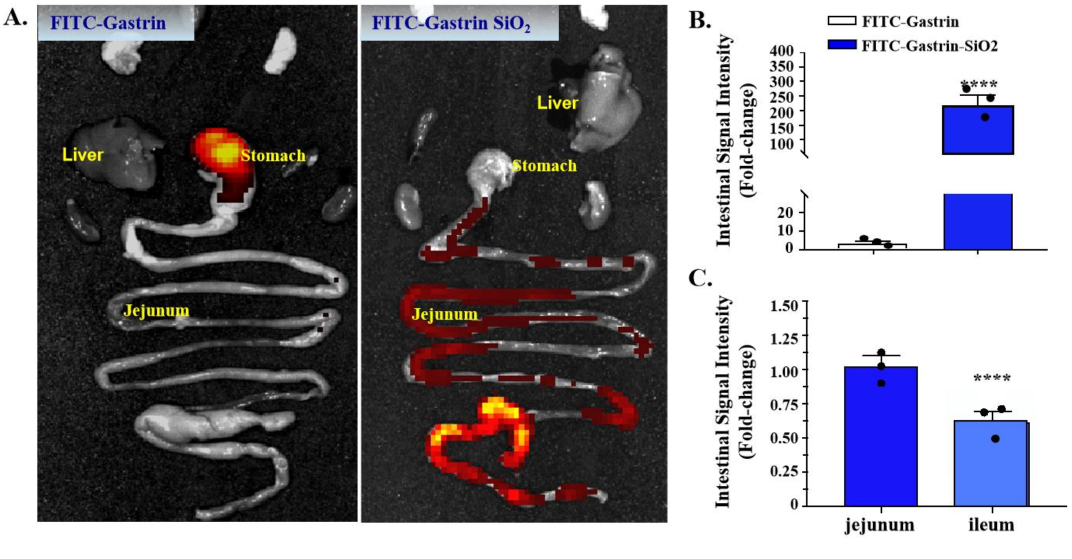

Figure S7. (A) Fluorescence is captured by fluorescence emission tomography. Gastrin-SiO_2 microspheres with FITC or Gastrin conjugated with FITC (control group) was gavaged into mice for 24 h. **(B)** Fluorescence of small intestine in the two groups of mice (n=3/group, ****P < 0.001 vs FITC-Gastrin). **(C)** Fluorescence of jejunum and ileum in FITC-Gastrin and Gastrin-SiO_2 microspheres-gavaged mice (n=3/group, ****P < 0.001 vs jejunum).

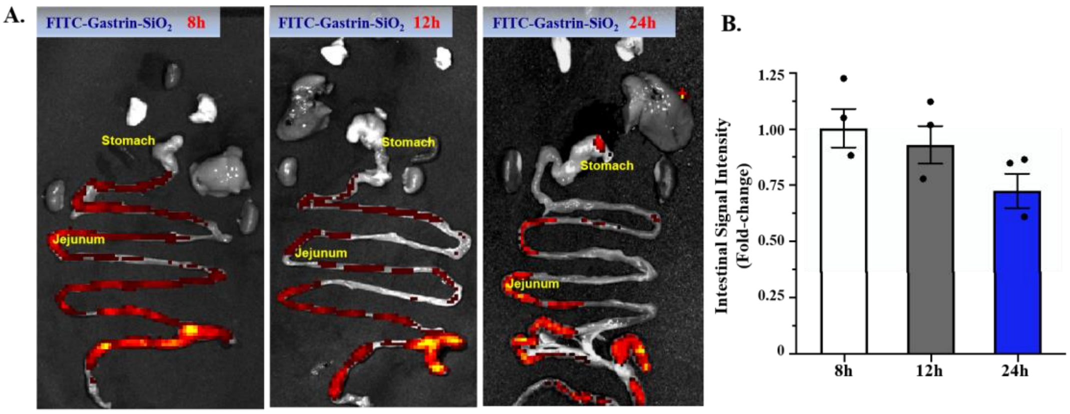

Figure S8. (A) Fluorescence is captured by fluorescence emission tomography at different times after gavage of FITC-Gastrin-SiO_2 microspheres **(B)** Fluorescence of the small intestine in the three groups of mice (n=3/group, **P < 0.01 vs 8 h).

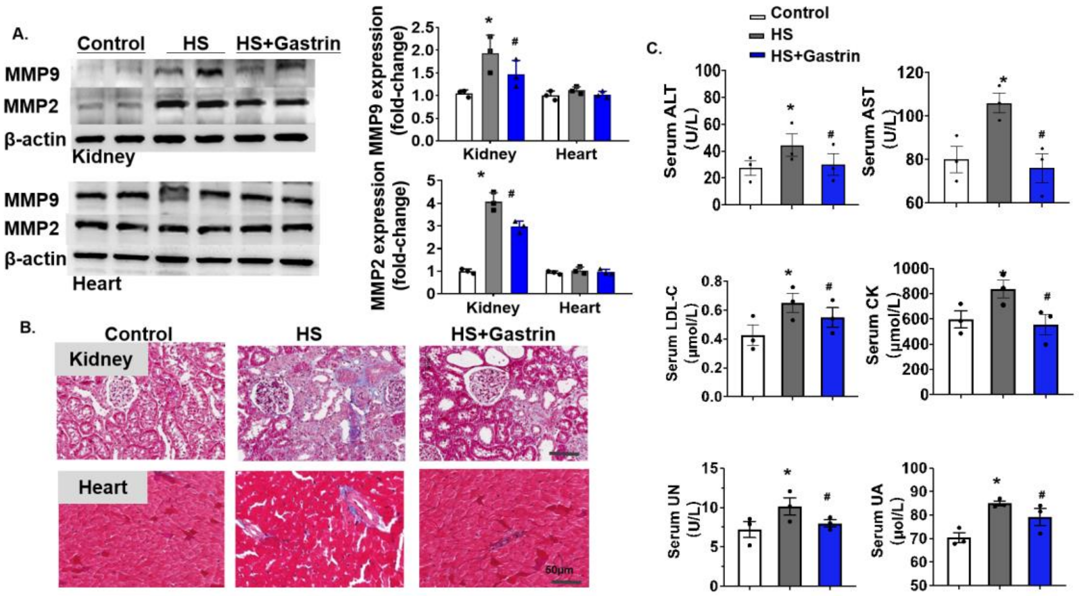

Figure S9.(A) MMP9 and MMP2 protein expressions were quantified by western blot

(n=4/group, *P<0.01 vs *Cckbr*$^{fl/fl}$ WT+NS (Control), $^{\#}$P<0.05 vs *Cckbr*$^{fl/fl}$ WT+HS, one-way ANOVA, Holm-Sidak test). **(B)** Masson-stained sections of the kidney and heart (scan bar is 50 μm). (C) Serum alanine transaminase (ALT), aspartate transaminase (AST), low density lipoprotein-cholesterol (LDL-C), creatine kinase (CK), urea nitrogen (UN), and uric acid (UA) in three groups of rats: Control=normal salt (0.49% NaCl), HS=high salt (8% NaCl), and HS+Gastrin-SiO2 microspheres (0.02 mg/kg).

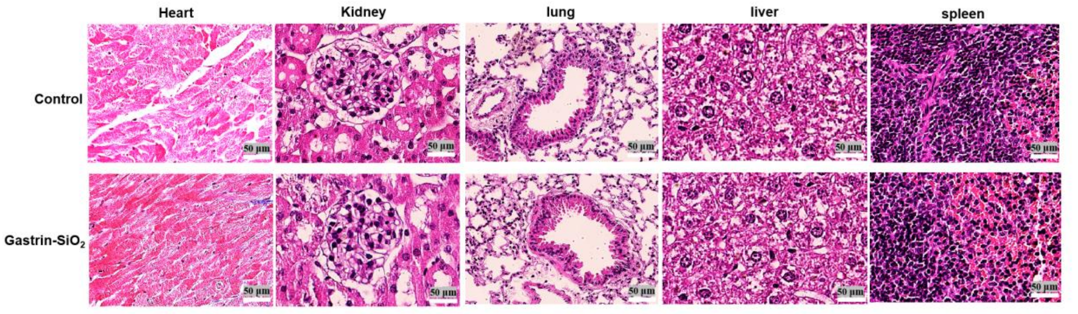

Figure. S10 Representative photographs of H&E staining of paraffin-embedded sections of different organs in Control and Gastrin-SiO_2 microspheres-gavaged groups.

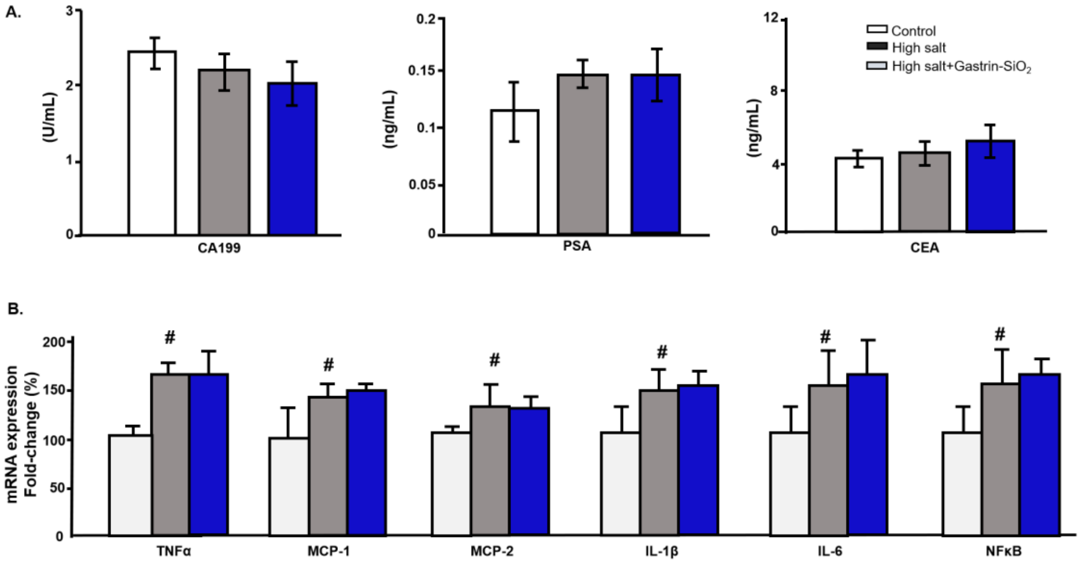

Figure S11. Serum markers of colon cancer and mRNA expressions of inflammation-associated genes in Gastrin-SiO_2 microspheres-treated mice. Serum concentrations of colon cancer markers CA199, PSA, and CEA **(A).** mRNA expressions of TNFα, MCP-1, MCP-2, IL-1β, IL-6, and NFkB in intestinal brush border membranes **(B).** (n=10/group, #P<0.05 vs Control, normal salt, 0.49% NaCl), one-way ANOVA, Holm-Sidak test. Data are presented as the mean±SE.

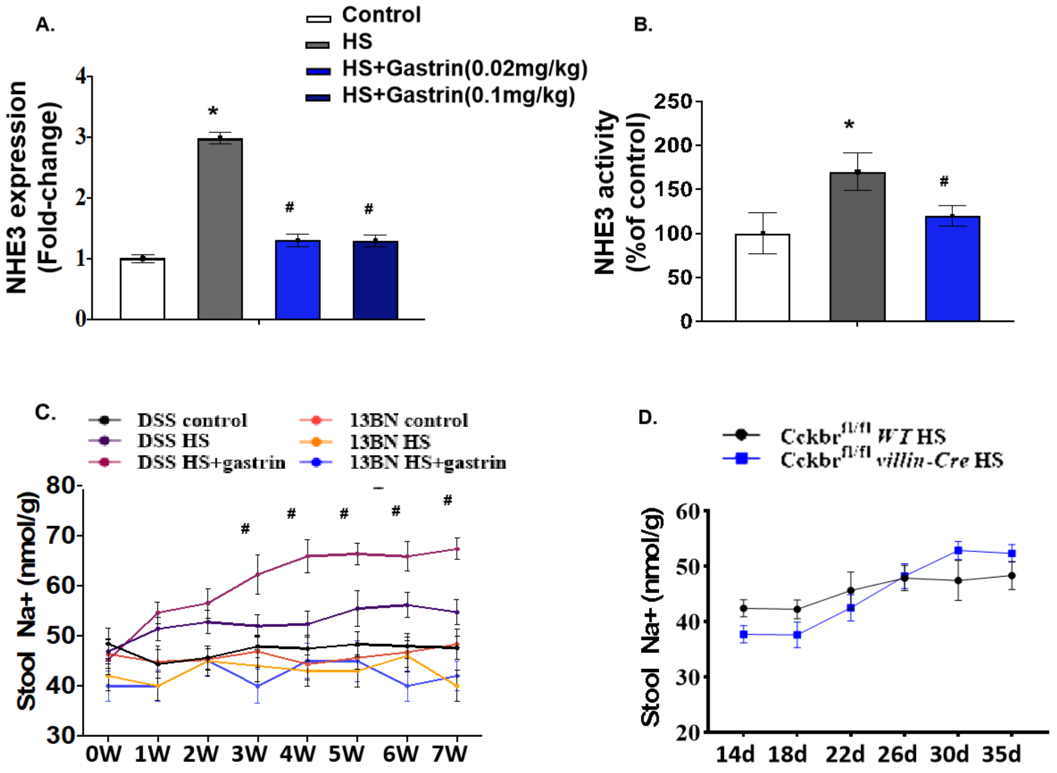

Figure S12. NHE3 expression**(A)** and activity **(B)** in the jejunum of Gastrin-SiO_2 microspheres-treated Dahl salt-sensitive rats (Control=normal salt (0.49% NaCl) diet, HS=high salt (8%) diet) ($^{*}P < 0.05$ vs Control,$^{\#}$ vs HS, one-way ANOVA, Tukey test. **(C)** Stool sodium in Dahl salt-sensitive (DSS) and $SS13^{BN}$ (13BN) rats fed Control=normal salt (0.49% NaCl) diet or HS=high salt (8%) diet. ($^{\#}$ vs others, one-way ANOVA, Tukey test). **(D)** Stool sodium in *Cckbr*$^{fl/fl}$ WT and *Cckbr*$^{fl/fl}$ *villin-Cre* (KO) mice fed high salt (HS) diet. WTN=*Cckbr*$^{fl/fl}$ WT mice fed NS diet, WTH=*Cckbr*$^{fl/fl}$ WT mice fed HS diet, KON=*Cckbr*$^{fl/fl}$ *villin-Cre* (KO) mice fed NS diet, KON=*Cckbr*$^{fl/fl}$ *villin-Cre* (KO) mice fed HS diet.

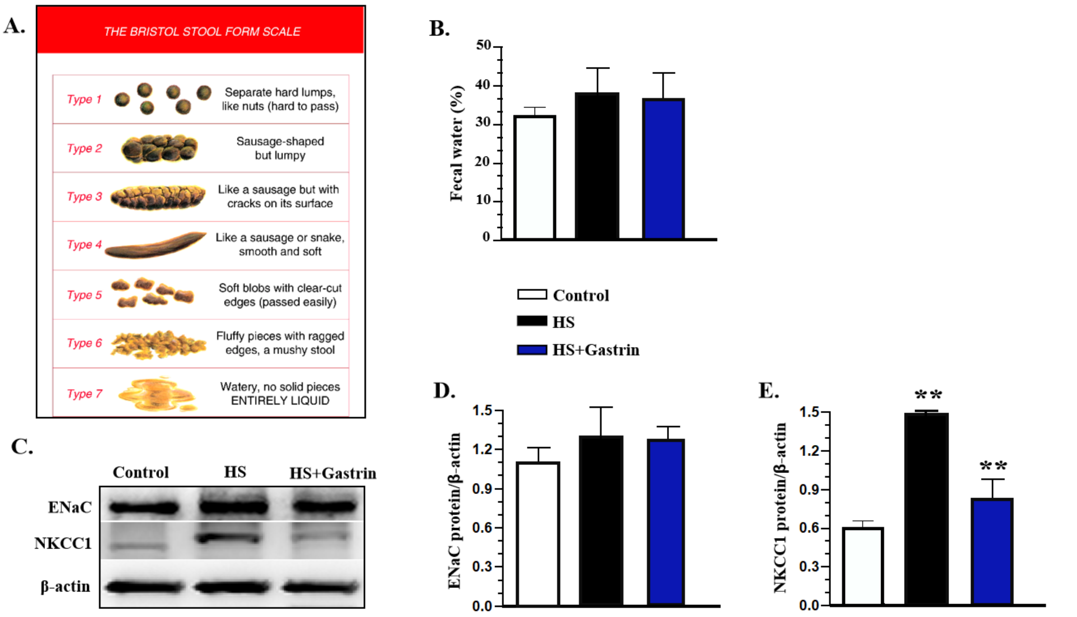

Figure S13. (**A**) Bristol Stool Chart showing Bristol grade 1-2 (dry and hard), 3-4 (normal), and 5-7 (diarrhea). (**B**) Effect of high salt (HS) diet with and without Gastrin-SiO_2 microspheres on fecal water content. (**C-D**) Effect of HS diet with and without Gastrin-SiO_2 microspheres on ENaC (**C, D**) and NKCC1 (**C, E**) proteins in small intestines, quantified by western blot (n=4/group, **P<0.01 vs others, one-way ANOVA, Holm-Sidak test). (Control = 0.49% NaCl diet, HS = 8% NaCl diet, Gastrin = Gastrin-SiO_2 microspheres (0.02 mg/kg)
